# Effects of school-based physical activity and multi-micronutrient supplementation intervention on growth, health and well-being of schoolchildren in three African countries: the *KaziAfya* cluster randomised controlled trial protocol with a 2 × 2 factorial design

**DOI:** 10.1186/s13063-019-3883-5

**Published:** 2020-01-06

**Authors:** Markus Gerber, Serge A. Ayekoé, Johanna Beckmann, Bassirou Bonfoh, Jean T. Coulibaly, Dao Daouda, Rosa du Randt, Lina Finda, Stefanie Gall, Getrud J. Mollel, Christin Lang, Kurt Z. Long, Sebastian Ludyga, Honorati Masanja, Ivan Müller, Siphesihle Nqweniso, Fredros Okumu, Nicole Probst-Hensch, Uwe Pühse, Peter Steinmann, Sylvain G. Traoré, Cheryl Walter, Jürg Utzinger

**Affiliations:** 1 Department of Sport, Exercise and Health, University of Basel, Basel, Switzerland; 2 Institut National de la Jeunesse et des Sports, Abidjan, Côte d’Ivoire; 3 Swiss Tropical and Public Health Institute, Basel, Switzerland; 4 University of Basel, Basel, Switzerland; 5 Centre Suisse de Recherches Scientifiques en Côte d’Ivoire, Abidjan, Côte d’Ivoire; 6 Unité de Formation et de Recherche Biosciences, Université Félix Houphouët-Boigny, Abidjan, Côte d’Ivoire; 7 Nelson Mandela University, Port Elizabeth, South Africa; 8 Ifakara Health Institute, Dar es Salaam/Ifakara, Tanzania; 9 Unité de Formation et de Recherche des Sciences et Technologies des Aliments, Université Nangui Abrogoua, Abidjan, Côte d’Ivoire

**Keywords:** Children, Côte d’Ivoire, Dual disease burden, Health, Multi-micronutrient supplementation, Placebo, Physical activity, South Africa, Tanzania, Well-being

## Abstract

**Background:**

In low- and middle-income countries, infectious diseases remain a key public health issue. Additionally, non-communicable diseases are a rapidly growing public health problem that impose a considerable burden on population health. One way to address this dual disease burden, is to incorporate (lifestyle) health promotion measures within the education sector. In the planned study, we will (i) assess and compare physical activity, physical fitness, micronutrient status, body composition, infections with soil-transmitted helminths, *Schistosoma mansoni*, malaria, inflammatory and cardiovascular health risk markers, cognitive function, health-related quality of life, and sleep in schoolchildren in Côte d’Ivoire, South Africa and Tanzania. We will (ii) determine the bi- and multivariate associations between these variables and (iii) examine the effects of a school-based health intervention that consists of physical activity, multi-micronutrient supplementation, or both.

**Methods:**

Assuming that no interaction occurs between the two interventions (physical activity and multi-micronutrient supplementation), the study is designed as a cluster-randomised, placebo-controlled trial with a 2 × 2 factorial design. Data will be obtained at three time points: at baseline and at 9 months and 21 months after the baseline assessment. In each country, 1320 primary schoolchildren from grades 1–4 will be recruited. In each school, classes will be randomly assigned to one of four interventions: (i) physical activity; (ii) multi-micronutrient supplementation; (iii) physical activity plus multi-micronutrient supplementation; and (iv) no intervention, which will serve as the control. A placebo product will be given to all children who do not receive multi-micronutrient supplementation. After obtaining written informed consent from the parents/guardians, the children will be subjected to anthropometric, clinical, parasitological and physiological assessments. Additionally, fitness tests will be performed, and children will be invited to wear an accelerometer device for 7 days to objectively assess their physical activity. Children infected with *S. mansoni* and soil-transmitted helminths will receive deworming drugs according to national policies. Health and nutrition education will be provided to the whole study population independently of the study arm allocation.

**Discussion:**

The study builds on the experience and lessons of a previous study conducted in South Africa. It involves three African countries with different social-ecological contexts to investigate whether results are generalisable across the continent.

**Trial registration:**

The study was registered on August 9, 2018, with ISRCTN. 10.1186/ISRCTN29534081.

## Background

Ensuring healthy lives and promoting well-being among children is a complex and challenging endeavour. Indeed, children’s health depends on cultural, environmental, genetic and socioeconomic factors as well as current living conditions and social and community networks [1]. In low- and middle-income countries (LMICs), infectious diseases remain a key public health issue, negatively impacting children’s physical and cognitive development [2]. For example, more than a billion people are infected with parasitic worms (helminths) [3]. Helminth infections can cause abdominal pain, anaemia and (bloody) diarrhoea and might impair cognitive and physical development [4], resulting in reduced fitness and work productivity [5]*.* Moreover, helminth infections can have a negative impact on a child’s nutritional status [6]. A deprived socioeconomic status (SES) of parents can put children at risk of malnutrition and growth retardation. Malnutrition has been found to be associated with stunting and poor cognitive development, thereby resulting in low IQ, cognitive delays and problems with motor development. This, in turn, can cause problems with a child’s ability to concentrate, process information and focus on school work [7]. Children from low SES families are also less likely to have access to health care or health insurance and are more prone to be absent from school, which may have negative consequences on their academic performance. These deficiencies can prevent school-aged children from realising their full potential and perpetuate a vicious cycle of poverty and poor health.

Non-communicable diseases (NCDs) are a rapidly growing public health problem that impose a considerable burden on population health [8]. New research revealed that African populations have moved towards a disease profile similar to Western countries, with increasing proportions of deaths attributed to chronic, lifestyle-related diseases [9] and overweight, replacing undernutrition as a risk factor [2, 10]. Consequently, children are at an increased risk of compromised health due to a dual burden of diseases, which may hamper their development and well-being [8, 11]. The drivers of this dual burden may relate to the shift in dietary consumption patterns and energy expenditure as these countries pass through rapid nutritional and epidemiological changes. This trend might also result from the effect that underlying micronutrient deficiencies have on childhood stunting and changes in body composition, leading to greater adiposity and possibly contributing to long-term risks of obesity [12–15]. This dual burden constitutes a challenge for health systems in Africa and elsewhere. Although many children are still affected by infectious diseases, at a young age, they may already have developed risk factors predisposing them to NCDs in early adulthood [16, 17].

Given that (i) childhood physical inactivity is an independent risk factor for NCDs, which can lead to poor health outcomes in later life [18–20] and that (ii) micronutrient status influences health and body composition and subsequently the development of obesity and obesity-related conditions [21], one plausible strategy is to focus on the promotion of physical activity and multi-micronutrient supplementation through school-based health promotion programmes. School-based physical activity interventions are worthwhile because a considerable amount of children’s daily physical activity is acquired during school hours [22]. Moreover, school-based physical activity programmes are generally effective in increasing physical activity and physical fitness in children and adolescents aged 6–18 years [23]. For instance, a randomised controlled trial with Swiss primary schoolchildren (first and fifth graders) showed that physical activity and fitness could be significantly improved with a 1-year, school-based, physical activity intervention, whereas adiposity could be decreased [24].

An attempt to increase health literacy in South African children from disadvantaged schools was undertaken in the ‘Disease, Activity and Schoolchildren’s Health’ (DASH) project [3]. The study primarily focused on the development of healthy school environments by implementing a series of clearly defined and standardised intramural measures. The developed intervention toolkit was pilot-tested among fourth graders in disadvantaged primary schools in Port Elizabeth, South Africa. The preliminary findings suggest that (i) the prevalence of parasitic worm infection was high in several schools [25]; (ii) children infected with soil-transmitted helminths had lower maximal oxygen uptake compared to their non-infected peers [26]; (iii) helminth infections and low physical fitness were significant predictors of low selective attention and poor academic achievement [27]; (iv) increased levels of physical activity were associated with a higher health-related quality of life (HRQoL) [28]; (v) increased physical activity levels were associated with lower risks of obesity and hypertension, but increased risk for soil-transmitted helminth infections [29]; and (vi) the DASH physical activity component resulted in small but significant decreases in the body mass index (BMI) of the children [30] and had a positive impact on academic performance within one year [31]. The intervention teaching material used in the DASH study was further elaborated and adapted and then pilot-tested among schoolchildren in grades 4–7 from two disadvantaged primary schools in Port Elizabeth. After the pilot-testing, final adaptations were made according to the feedback of school teachers, teacher-coaches and students.

The present study will build on and expand DASH by implementing this school-based health promotion programme in two other African countries and by providing multi-micronutrient supplementation. Placing an additional emphasis on multi-micronutrient supplementation is justified on multiple grounds. First, a recent meta-analysis concluded that helminth infections and micronutrient deficiencies are highly prevalent in LMICs. Second, a strong relationship exists between helminth infections and serum retinol in school-aged children. Third, micronutrient-supplementation randomised controlled trials (RCTs) showed a modest, but significant protective effect on helminth infection and reinfection rates [32]. Fourth, periodic deworming has not translated into the expected health gains according to recent systematic reviews and meta-analyses [33, 34]. One reason for this may be the concurrent problem of malnutrition and underlying micronutrient deficiencies, which makes it difficult for children to compensate for delays in growth and development that resulted from infectious diseases. Thus, multi-micronutrient supplementation might make deworming more effective in terms of a positive health impact. Fifth, as shown in previous studies [35, 36], multi-micronutrient supplementation might result in a decrease in fat mass and increased lean body mass.

### Specific aims of the study

There are three interrelated specific aims that will be addressed in the planned study:

- To assess and compare physical activity, physical fitness, micronutrient status, body composition, infections with soil-transmitted helminths, *S. mansoni*, malaria, inflammatory and cardiovascular health risk markers, cognitive function, HRQoL, and sleep in schoolchildren in Côte d’Ivoire, South Africa and Tanzania.
- To determine the bi- and multivariate associations that exist among physical activity, physical fitness, micronutrient status, body composition, infections with soil-transmitted helminths, *S. mansoni*, malaria, inflammatory and cardiovascular health risk markers, cognitive function, HRQoL, and sleep in schoolchildren in the three study countries.
- To examine the effects of a school-based health intervention (physical activity, multi-micronutrient supplementation or both) on physical activity, physical fitness, micronutrient status, body composition, infections with soil-transmitted helminths, *S. mansoni*, malaria, inflammatory and cardiovascular health risk markers, cognitive function, HRQoL, and sleep in schoolchildren in the three study countries.

Additionally, secondary aims are to determine how the intervention is perceived by the school principals, teachers, parents and children, and whether or not the teachers can be empowered to implement the teaching material successfully by themselves. The study also has the potential for highlighting how micronutrient deficiencies may contribute to the observed increases in obesity prevalence, which is on the rise in some African countries.

Specific hypotheses for each of the outcome variables were formulated, hereby taking into account existing evidence from previous studies, mostly carried out with children living in Western societies. These hypotheses are described in detail in the supplementary material (see Additional file 1).

## Methods/Design

### Study design

**Fig. 1:**
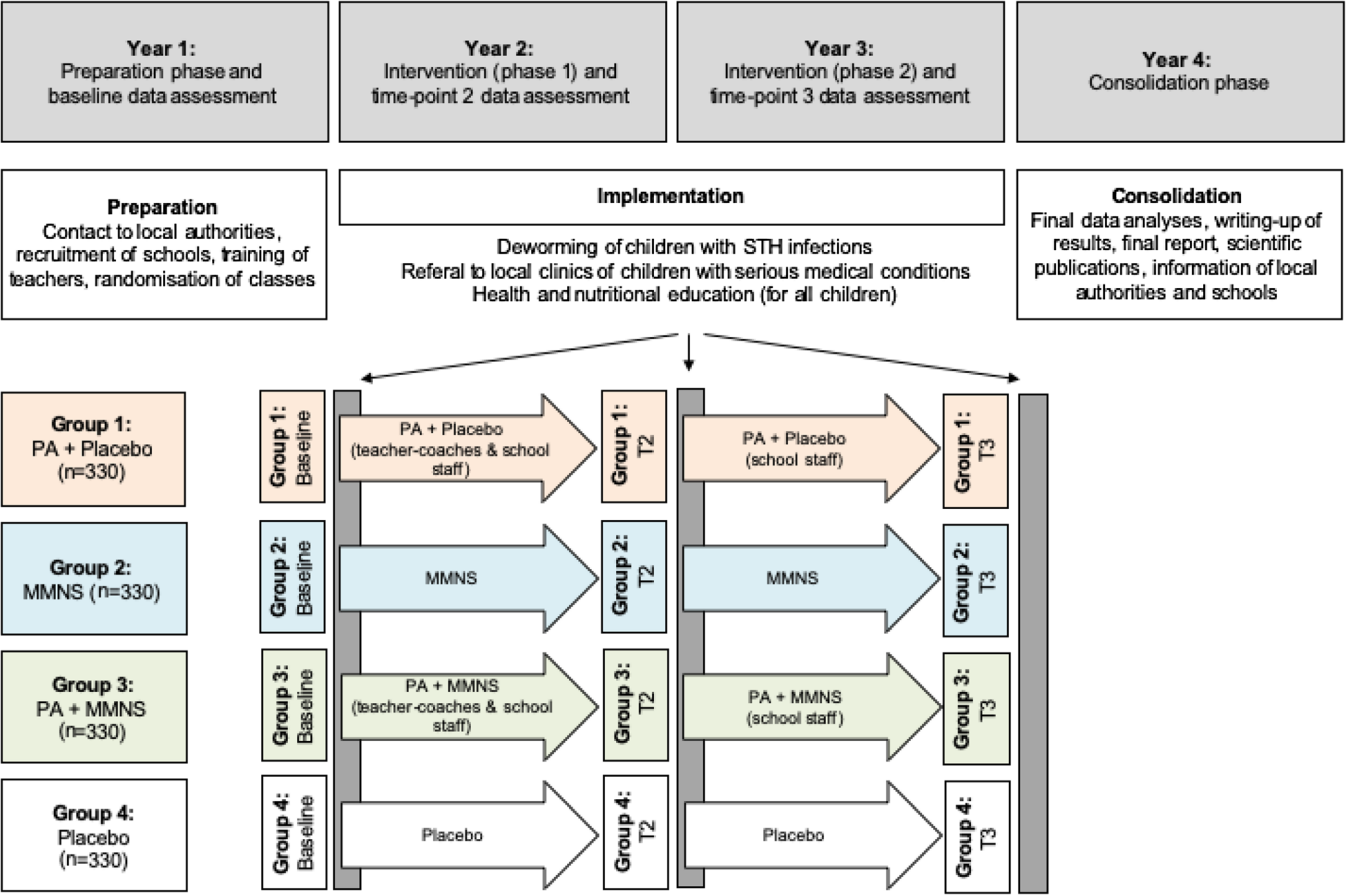
Overview of the planned study design (*Note*. MMNS = multi-micronutrient supplementation; PA = physical activity; STH = soil-transmitted helminth; T2 = 9-month follow-up; T3 = 21-month follow-up). All children receive health and nutritional education during the 2-year study period 4

We assume that no interaction occurs between the two interventions (physical activity and multi-micronutrient supplementation). The study is designed as a cluster randomised, placebo-controlled trial [37] using a 2 × 2 factorial design to assess the effect of physical activity and multi-micronutrient supplementation on children’s growth, health and well-being (Fig. 1). Data will be assessed at three time points (baseline and 9 months and 21 months after the baseline assessment). At baseline, children from grades 1–4 (most of them aged 6–10 years) will be recruited. The intervention will span two school years. The project officially started in January 2018. After 9 months of preparation and obtaining ethical approval from the relevant authorities, interventions have been launched in 2019. The project will last until December 2021 (see SPIRIT Flow Chart in Additional file 2).

The study will be carried out in primary schools in the area of Taabo in south-central Côte d’Ivoire, in Port Elizabeth in the Eastern Cape Province of South Africa, and in Ifakara in the Kilombero district of Tanzania. To obtain an adequate sample size for overweight/obese children, the intervention will not take place in the most remote areas but rather in villages that are located in rural (Côte d’Ivoire and Tanzania) or peri-urban (South Africa) settings in the three study countries.

In each country, we aim to recruit 1320 children from public school classes. Classes will be randomly assigned to one of four interventions (for more details, see the next section). To ensure allocation concealment, the four treatment arms will be determined by a computer-generated code after the baseline assessment. The four intervention arms are (i) physical activity; (ii) multi-micronutrient supplementation; (iii) physical activity plus multi-micronutrient supplementation; and (iv) no specific interventions, which will serve as the control. Based on the four intervention arms, the main and interaction effects of the two intervention components (physical activity and multi-micronutrient supplementation) will be examined.

We will provide a placebo product to all children who do not receive multi-micronutrient supplementation. For physical activity, controlling with a placebo is not feasible. Thus, classes not involved in the physical activity intervention will follow their routine lesson plans so that, during the intervention phase, all students will have similar amounts of contact with schoolmates and teachers. To minimise subjective biases, teachers and local study personnel will be blinded with regard to the multi-micronutrient supplementation or placebo tablets.

In case of missing values (e.g. when children drop out during the intervention), all analyses will be performed with and without intention-to-treat [38]. After a thorough dropout analysis, a decision will be made on the most suitable method to use to analyse the intention-to-treat effects (e.g. imputation of missing values) [39].

### Participants and procedures

All children will be recruited in the schools involved in the project. School authorities will be contacted first. Contact with schools is made through the school principals. School principals will be informed about the objectives, procedures and potential risks and benefits of the study. Based on this information, the principals can state their interest in being part of the project.

To achieve at least small effects (f = 0.10) in the primary outcomes (physical activity and micronutrient deficiency) and to take into account the children’s weight status (underweight, normal weight, or overweight/obese), power calculations indicate that a total sample of 1096 children is needed per study site (calculations based on G*power 3.1: alpha error probability = 0.05, power = 0.80, number of groups = 12 (2x2x3: physical activity intervention: yes/no, multi-micronutrient supplementation: yes/no; weight status: underweight, normal weight, or overweight), number of measurements = 3). Assuming a yearly dropout-rate of 10%, the targeted sample size is 1320 children per country at baseline. Thus, in each country, approximately 330 students will be assigned to one of the four intervention arms.

**Fig. 2:**
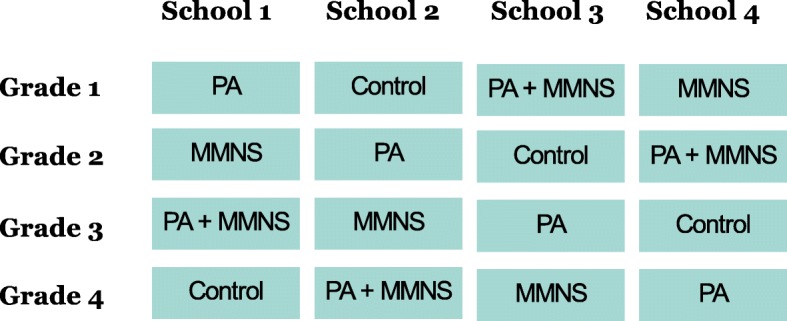
Assignment of classes to conditions via stratification to ensure that each intervention arm is represented at each grade across all schools (*Note*. PA = physical activity; MMNS = multi-micronutrient supplementation)

After having identified suitable schools, we will proceed in two steps to allocate classes to the four intervention arms. First, we develop a stratification table to ensure that each intervention arm will be represented in every grade across all schools. We then randomly assign the schools to the different strata, as shown in Fig. 2. If a school has several classes per grade, the classes will be randomly selected. Given that our intervention takes place in a school setting, is implemented during official class hours, is integrated in the current curriculum, and is provided by the class teachers, we have no choice other than to randomise the participants in clusters (classes). A division of classes (in order to allow individual randomisation) would affect the everyday school life in a negative way and would be considered interruptive by the teacher staff. As a consequence, our intervention would not be acceptable to the school principals and education authorities.

Before baseline data assessment, written informed consent will be sought from the parents/guardians of the children. In line with the Ottawa Statement [40], despite cluster randomisation, we will ask each research participant and his/her parents guardians for informed consent before the children and their parents guardians know to which cluster they will be allocated. Research assistants will explain the purpose and procedures of the study, the expected duration, potential risks and benefits and any discomfort it may entail for the children. The parents/guardians will be provided with a participant information sheet and a consent form describing the study and providing sufficient information to make an informed decision about whether or not to participate. For illiterate parents, the information sheet will be read aloud and, if necessary, an oral translation of the information sheet into any of the local languages will be provided. Parents/guardians will be informed that participation is voluntary, data will be handled confidentially, and withdrawal from the study can occur anytime without further obligation, and that withdrawal of consent will not have any negative consequences except forgoing the potential benefits of the allocated intervention. After having obtained written informed consent, the parents/guardians will be asked a few specific questions regarding the families’ SES, and the dietary intake information, sleep and physical activity behaviour of the children. Additionally, oral assent will be sought from children before the start of the study.

### Criteria for inclusion/exclusion

School eligibility criteria include (i) public schools from disadvantaged areas; (ii) facilities for the implementation of physical education lessons (free space: lawn, sand or concrete ground); and (iii) not engaged in any other research project or clinical trial or located in areas where governmental nutrition interventions take place.

To be included in the data assessment, children must accomplish the following inclusion criteria: (i) attend grades 1–4 at baseline; (ii) aged 6–12 years at baseline; (iii) have written informed consent from their parents/guardians; (iv) not participating in other research projects or clinical trials; (v) not participating in any food/nutritional programme; and (vi) not suffering from clinical conditions that prevent participation in physical activity, as determined by qualified medical personnel.

Children will be excluded from data analyses if (i) they have a congenital or acquired alteration of the gastro-intestinal tract, which could impair absorption of the multi-micronutrient supplements; (ii) they participated in food/nutritional programmes in the past 6 months, and hence, received regular vitamin and mineral supplements; (iii) their parents/guardians did not provide written informed consent; and (iv) the children denied oral assent.

Since some parts of the intervention are integrated in the compulsory school curriculum, children will be automatically exposed to physical activity and health/nutrition education intervention. In fact, physical education is foreseen in the curriculum in all three study countries, not as a separate subject but as part of other (broader) subjects. For instance, in South Africa, physical education is part of the ‘life orientation’ subject. Nevertheless, physical education is not implemented as it should be in any of the involved countries. Typically, this slot is used to increase learning time for academic subjects. Therefore, our intervention aims to ensure that the time allocated to physical education in the curriculum is used appropriately and filled with meaningful content.

In the event that qualified medical personnel identify children during the baseline data assessment as having a clinical condition that prevents their participation in physical activity, we will contact the school principal and suggest that these children be removed from the physical activity intervention. Moreover, as multi-micronutrient supplementation and deworming are additional components, children and their parents/guardians who did not provide informed consent can decide whether or not the child should participate in the multi-micronutrient supplementation and/or deworming. Finally, all children have the possibility to withdraw from the study anytime without consequences from these two additional components.

### Intervention and control conditions

Participants allocated to the physical activity intervention will receive the so-called *KaziKidz* physical activity component (see: www.kazibantu.org). Of note, regular physical activity opportunities are incorporated into the main school curriculum, including daily in-class activity breaks as well as one weekly 40-min playful physical education lesson and one 40-min moving-to-music lesson. These measures are designed towards improving children’s physical activity levels and positively affecting their school satisfaction and psychosocial well-being. The intervention materials presented above were pilot-tested in 2015 and 2016 in the DASH study with fourth grade children from disadvantaged schools in Port Elizabeth, South Africa. Qualitative data revealed that the physical activity materials were well received at the pilot schools.

Participants allocated to the multi-micronutrient supplementation condition will receive a daily chewable tablet containing vitamins and trace elements. The multi-micronutrient supplement is provided free of charge by DSM Nutritional Products Ltd. (Basel, Switzerland; see: www.dsm.com). The exact composition of the multi-micronutrient supplement is summarised in Table 1. During school days, the supplement is taken at schools under the direct supervision of a teacher. To avoid the risk that supplements are exchanged between students or given to other family members during weekends or public holidays, no supplements will be provided on non-school days.

**Table 1:** Composition of the multi-micronutrient supplement

No.	Nutrient	Average per 1 tablet
1	β-carotene (as BetTab 20%S)	3.6 mg
2	Vitamin D	400 IU/10 mcg
3	Vitamin E	9 mg TE
4	Vitamin K	30 mcg
5	Vitamin C	60 mg
6	Vitamin B1 Thiamine	1.1 mg
7	Vitamin B2 Riboflavin	1.3 mg
8	Vitamin B6 Pyriodoxine	0.5 mg
9	Vitamin B12	1.2 mcg
10	Folic acid	200 mcg
11	Niacinamide	8 mg
12	Iron (added as Fe-EDTA)	8 mg
13	Zinc (added as zinc oxide)	5 mg
14	Selenium (added as sodium selenite anhydrous)	20 mcg
15	Iodine (added as potassium iodate)	100 mcg

*Notes.* Ingredients 1–15 are nutrients and will be produced with an overage to ensure required amounts during the shelf life

Inactive ingredients: sugar, citric acid, sorbitol, non-nutritive sweetener, flavour

Placebo tablets do not contain nutrients, but colorants will be added

Dosage and directions: one tablet daily during the first school lesson

The tablets will be stored in a climate-controlled storage room at the respective research institution and will be provided to school on a fortnightly basis. At school, the tablets will be stored in locked cupboards in the teachers’ room

Participants allocated to the physical activity plus multi-micronutrient supplementation condition will receive both intervention measures, as described above. Children who do not receive multi-micronutrient supplementation (physical activity only and control conditions) will receive a placebo product. Thus, during school days, they will receive a daily chewing tablet, similar in taste and appearance to the multi-micronutrient supplementation and administered to the children from identical packages. The tablet will not contain any macronutrients or micronutrients, except sugar, citric acid, water and artificial flavour (orange) to mask the taste and to ensure similar appearance.

### Complementary interventions for all participating children

Independently of the study arm allocation, children diagnosed with helminth infections will receive deworming medication after each data assessment. The treatment strategy chosen for each school will follow national and international guidelines, including recommendations by the World Health Organization (WHO) [41]. In brief, in schools where the infection prevalence of soil-transmitted helminths is below 20%, infected children will be treated individually; in schools where the infection prevalence of soil-transmitted helminths is between 20% and 50%, all children will be treated once a year; and, finally, in schools were the prevalence of soil-transmitted helminth infection is 50% and above, mass treatment will be carried out twice a year. We will either administer a single 400-mg oral dose of albendazole or a single 500-mg oral dose of mebendazole. Additionally, children infected with *S. mansoni* will receive praziquantel (single 40 mg/kg oral dose).

All children will additionally benefit from health and nutritional education lessons. The developed *KaziKidz* teaching material includes a series of classroom-based health education lessons to increase awareness of helminth infections, how to prevent helminth infections (e.g. hygiene behaviour, adequate sanitation habits and the importance of consuming clean water and healthy food). Likewise, nutritional education lessons aim at highlighting the importance of healthy nutrition.

### Education and training of the teachers

The initial experiences in the DASH study showed that teachers needed considerable support for the implementation of the physical education lessons. In the planned study, during the initial phase, the intervention will be carried out in close collaboration among the teacher-coach, teachers, and school staff to allow sufficient time for capacity building and to empower schools to incorporate the programme in their given structures and to implement the programme with their own resources. Hence, teachers will be assisted by a teacher-coach during the first year of the intervention, whereas during the second year, the physical activity intervention will be carried out by the teachers themselves without external assistance.

### Monitoring of compliance with intervention implementation

One of the specific aims of the study is to obtain new insights regarding the possibility to empower schools to implement sustainable health-promotion measures, including an appraisal of the implementation quality of health-promotion measures, if carried out under the direction of the schools themselves. Using both quantitative and qualitative research methods, such as the systematic observation of lessons; (semi-)structured interviews with school principals and parents; and focus group discussions (FGDs) with teachers, teaching staff members, and children, the study will shed light on the quality of the implementation and sustainability of the proposed health promotion measures, as well as factors affecting the quality of the implementation and the sustainability of the programme. Thereof, important insights will result with regard to the training of the teachers and school staff, as well as the support needed by the schools to incorporate health-promotion measures in the existing structures.

### Data collection and measures

Data collection will take place in the schools. Data assessment procedures will be based on a series of standardised, validated and quality-controlled tools. The same methods will be employed at each measuring occasion (except for the parent questionnaires, which will only take place during the baseline assessment). The collected data will be double-entered and validated using EpiData (version 3.1) and merged into a single database.

The following parameters are defined as equivalent primary outcomes: (i) physical activity (7-day accelerometry), and (ii) multi-micronutrient status (vitamin A, vitamin D, transferrin and zinc). In our study, physical activity is assessed via self-reports and 7-day accelerometry. Although both methods have advantages and disadvantages [42, 43], many researchers consider accelerometer-derived data as the most appropriate way to validly assess physical activity [44]. This particularly applies to children, where the assessment of self-reported physical activity is complicated by the fact that some cognitive functions are not yet fully developed. Multi-micronutrient status, per definition, is a multi-dimensional construct. Given that our supplement contains several (nutritional) ingredients, one can expect that the product will be effective in terms of multiple outcomes. As highlighted by Bailey et al. [45], iron, iodine, folate, vitamin A and zinc deficiencies are the most widespread multi-micronutrient deficiencies and are common contributors towards poor growth, intellectual impairment, and increased risk of morbidity. Making a decision about whether one nutritional indicator is more important than another is arbitrary and cannot be convincingly substantiated. Therefore, we consider vitamin A, vitamin D, transferrin and zinc as equivalent primary outcomes.

Table 2 provides an overview of all types of data to be collected and the specific parameters that will be assessed either as primary outcomes, secondary outcomes, moderators or control variables.

**Table 2:** Overview of parameters assessed in the planned study

Clinical examinations	
Disease history of children and parents/guardians	
Subjective health complaints (15 items)	
Blood pressure (SBP, DBP)	
Blood testing	
Haemoglobin concentration (Hb)	
Blood lipids (TC, HDL-C, LDL-C, TG, Non-HDL, C-HDL ratio)	
Blood glucose (HbA1c)	
Micronutrient status (vitamin A, vitamin D, zinc, transferrin)	
Cytokines (IL-6)	
Leptin	
Anthropometric measurements	
Body weight and height	
Body composition (body fat)	
Waist-to-hip ratio	
Body mass index	
Parasitological examinations	
Soil-transmitted helminths (*Ascaris lumbricoides*, hookworm, *Trichuris trichiura*)	
*Schistosoma mansoni*	
Cognitive function and academic performance	
Flanker task (executive function)	
School grades	
Student survey	
Socio-demographic background (sex, ethnicity, home language)	
Self-reported physical activity	
Health-related quality of life (KIDSCREEN-10)	
Perceived stress (1 item)	
School satisfaction (1 item)	
Perceived academic competence (1 item)	
Sleep (6 items)	
Objective assessment of physical activity	
7-day actigraphy	
Fitness testing	
20-m shuttle run test (cardiorespiratory fitness)	
Grip strength test (upper body strength)	
Parental survey	
Family socioeconomic status	
Dietary intake information (food frequency questionnaire)	
Food insecurity	
Sleep	
Other variables	
Country, school, grade, class, distance/traveling to/from school	

#### Clinical examination

A research assistant will assess detailed disease history in a face-to-face interview. Features of disease history will focus on fevers, abdominal pain, change in bowel movements, diabetes, and psychosomatic symptoms. Additionally, a qualified nurse will conduct an abdominal examination.

For the detection of hypertension, the blood pressure of each child will be taken three times after the child has been resting for approximately 5 min, with a 1-min rest in between the assessments. An Omron M3® digital blood pressure monitor (Omron Healthcare Europe; Hoofddorp, The Netherlands) will be used. A cuff size appropriate to the arm circumference of the child will be chosen.

#### Blood testing

Capillary blood will be collected for haematological analyses. The child’s finger will be pricked once (or if necessary twice) to collect approximately 10 blood drops. Haemoglobin (Hb), blood lipid and blood glucose analyses will be performed on the spot (further details are provided below), with rapid finger prick malaria tests being done in Côte d’Ivoire and Tanzania. All tests will be performed with the same measurement devices and are carried out by trained research assistants. Testing cassettes will be disposed immediately after completion of the analyses.

##### Haemoglobin

For the detection of anaemia, Hb concentration will be measured once with a HemoCue® Hb 301 system according to the manufacturer’s instructions (HemoCue AB; Ängelholm, Sweden).

##### Blood glucose

For the measurement of glycated haemoglobin (HbA1c) level, a point-of-care (POC) instrument employing the Afinion test (Alere Technologies, Abbott; Wädenswil, Switzerland) will be used. Notably, the HbA1c level reflects the average plasma glucose concentration levels over the previous 8–12 weeks before measurement with no prior fasting required.

##### Blood lipids

For the assessment of blood lipid profiles (total cholesterol (TC), low-density-lipoprotein cholesterol (LDL-C), high-density-lipoprotein cholesterol (HDL-C) and triglycerides (TG), capillary samples for blood lipid will be analysed by the Afinion test (Alere Technologies, Abbott; Wädenswil, Switzerland). One drop of blood will be taken up by the test strip and read by the machine. Children will be instructed to fast during the 3 h prior to the data assessment.

##### Micronutrient status, inflammatory cytokines and leptin

The finger prick technique will also be used to prepare dried blood spots to assess children’s micronutrient status/deficiencies (concentrations of vitamin A, vitamin D, zinc and transferrin) and to determine the inflammatory cytokine (IL-6) and leptin concentrations. Blood drops will be collected on a filter paper for further examination in a specialised laboratory. Dried blood spot samples on the filter paper will be shipped to the Global Clinical and Viral Laboratory (Durban, South Africa). The laboratory was involved in the WHO SAGE study [46] and is a partner institution of the Global Health Biomarker Lab at the University of Oregon, USA.

#### Anthropometric measurements

##### Body weight, body height and body composition

Body composition will be assessed via bioelectrical impedance analysis (BIA) with a wireless body composition monitor (Tanita MC-580, Tanita Corp.; Tokyo, Japan). The participants will be asked to fast for 3 h before the data assessment, to void their bladder immediately before the assessment, and to wear only light sport clothing (≤1 kg). Participants will be asked to stand barefoot on the metal plates of the machine, while being guided by the research assistant to ensure optimal contact according to the device manufacturer’s instructions. The MC-580 is also able to assess body weight, which will be measured to the nearest 0.1 kg. With shoes off, each child will stand against a stadiometer with the back erect and shoulders relaxed. Body height will be taken to the nearest 0.1 cm. Sex-specific height or length-for-age and weight-for-age *z* scores will be computed from the CDC/WHO growth reference data [47].

#### Parasitological examinations

For parasitological examinations, a researcher will visit the schools and distribute pre-labelled plastic containers to each class for the children to take home and use to collect a stool sample. These plastic containers will be returned to the research assistant in the morning of the following day. Further visits may be required to catch up with absent children. To reduce discomfort, paper bags are provided along with the containers. All stool samples will be processed on the day of collection at the study site.

Parasitic infections to be detected with the Kato-Katz technique [48] include the three main soil-transmitted helminths (*Ascaris lumbricoides*, hookworm and *Trichuris trichiura*), and *Schistosoma mansoni*. In brief, stool samples (at least 10–15 g) will first be visually examined for the presence of blood, mucus and diarrhoea. Second, duplicate 41.7 mg Kato-Katz thick smears will be prepared from each stool sample [48]. For quality control, a random sample of 10% of the Kato-Katz slides will be re-examined by a senior technician. In case of discordant results, the slides will be read a third time, and the results will be discussed among the technicians until agreement has been reached [49, 50]. Parasitological status will be established in terms of prevalence and intensity of infection with individual helminth species, and the extent of multiparasitism will be determined.

#### Cognitive function and academic performance

##### Executive function

Inhibitory control is a core component of executive function and will be assessed with a computer-based version of the Flanker task [51], a standardised psychological test [52] suitable for repeated measures [53]. The task requires participants to respond to the direction of a centrally presented target stimulus, while flanking stimuli are facing in the same (congruent trials) or opposite direction (incongruent trials). Performance is assessed by calculating the mean reaction time for correct responses as well as mean accuracy separately for different trial types. Congruent trials are a measure of basic processing speed and attention, whereas incongruent trials assess selective attention and inhibitory control.

##### Academic performance

In cooperation with the schools, the end of year marks will be obtained from the following subjects: school/home language, first additional language, mathematics and life orientation. The sum-score of the four subjects will be used to estimate a child’s overall academic achievement.

#### Student survey

##### Self-reported physical activity

Two self-report instruments will be used to assess physical activity in the present study [42, 54]. The first instrument is a single-item tool taken from the HBSC survey. The exact wording of this item is as follows: ‘Physical activity is any activity that increases your heart rate and makes you get out of breath some of the time. Over the past 7 days, on how many days did you engage in such activity?’ Answering options range from 1 to 7 days [55]. A similar item has been used in previous research enrolling children [56–58], including the DASH study in South Africa [28].

The second instrument is the Physical Activity Questionnaire for Children (PAQ-C), a 9-item instrument specifically designed for school-aged children [59]. Due to the limited age of our sample and time constraints, only five items will be included. The PAQ-C consists of a 7-day recall that provides a summary physical activity score derived from several items, which are each scored on a 5-point Likert-scale (from 1 to 5). Items included in the present study refer to physical activity accumulated during physical education, recess, after school, in the evening and on weekends. Previous research has shown that the PAQ-C has acceptable reliability and convergent validity [60–62]. Furthermore, cut-off values have been established which are suitable to distinguish between those children who accomplish the recommended levels of physical activity (≥60 min of moderate-to-vigorous physical activity (MVPA) per day) and children who do not meet these criteria [63].

##### Health-related quality of life

The KIDSCREEN-10 will be used to assess children’s HRQoL. The KIDSCREEN-10 proved to be a valid instrument to assess the psychosocial health of children aged 8–18 years in different countries [64]. The construct validity of the KIDSCREEN instrument has been documented in an African context [65]. Moreover, we have used the KIDSCREEN in the DASH study, where we found satisfactory psychometric properties of this instrument [28]. The KIDSCREEN-10 consists of 10 items, which can be used to build an overall HRQoL index. Following recommended procedures, item scores first will be summed up to obtain raw scores and then will be transformed into Rasch person parameter estimates using the available SPSS software version syntax for each dimension [66]. These steps will result in T-values with a scale mean of 50 and a standard deviation (SD) of 10. Higher mean scores generally reflect higher HRQoL. To be classified as ‘normal’, the threshold chosen by the KIDSCREEN developers was the mean, plus or minus half a SD. The KIDSCREEN scores can be compared with the norm scores of an international survey sample of 5754 European children, stratified by sex.

##### Stress, school satisfaction and perceived academic competence

Perceived school-related stress, satisfaction with school and perceived academic competence will be assessed with three items from the HBSC survey. The stress measure has been used previously to show evidence for the stress-buffering effects of physical activity in European youngsters [58]. To assess school-related stress, students are asked how pressured they feel by the schoolwork they must pursue. Possible answers are as follows: not at all, a little, some, and a lot. To measure school satisfaction, students respond to the question of how they feel about school at present. Possible answers are as follows: ‘I like it a lot’, ‘I like it a bit’, ‘I don’t like it very much’, and ‘I don’t like it at all’. Finally, perceived academic performance is assessed with the following question: ‘In your opinion, what does your class teacher(s) think about your school performance compared to your classmates?’ Possible answers are as follows: ‘much better than classmates’, ‘better than classmates’, ‘similar/same as classmates’, and ‘worse than classmates’.

##### Sleep

To assess sleep quality, questions from the Pittsburgh Sleep Quality Index (PSQI) [67] will be adapted. To screen for sleep disturbances, the three items of the Insomnia Severity Index [68] will ask about difficulty falling asleep, staying asleep and waking up too early in the morning. Evidence for the reliability and validity of this measure has been provided previously [69]. To assess further information about sleep quality and daytime functioning, children will be invited to rate their overall sleep quality and to report how restored they feel in the morning, how tired they feel during the day and how exhausted they feel in the evening. Children will also provide basic information about their sleep environment (e.g. room, type of bed, and people sleeping in the same room/bed).

#### Actigraphy

Objective physical activity will be assessed with an accelerometer device (Actigraph wGT3x-BT; Shalimar, FL, USA). The devices will be worn around the hip for 7 consecutive days to assess a full week, with a sampling epoch of 15 s [70]. Time per day spent in moderate physical activity (MPA; ≥3 metabolic equivalents of task (MET)) and vigorous physical activity (VPA; ≥6 MET) will be determined based on the raw accelerometry counts and the ActiLife® computer software (Actigraph; Shalimar, FL, USA), with cut-off values derived from Freedson et al. [71]. Of note, the ActiGraph accelerometers have been validated for children [72, 73].

#### Fitness testing

##### Cardiorespiratory fitness

The children’s cardiorespiratory fitness will be measured with the 20-m shuttle run test [74], which is part of the Eurofit fitness testing battery [75]. Before the start of the test, all children will be told to indicate any body discomfort and anyone who feels unwell or uncomfortable will not take part in the test. The pre-recorded sound signals will be played to the children, and they will be able to do a trial run of 2 intervals (40 m) under the supervision of a research staff member. Once children are familiar with the test procedures, they will be asked to run back and forth on the 20-m flat course (marked with colour-coded cones) in groups of 10–15 children, following the pace of the sound signals. Starting with a running speed of 8.5 km/h, the frequency of the signal increases every minute by 0.5 km/h. When a child fails to follow the pace in two consecutive intervals, the stage and the distance completed fully will be recorded. The age of the participating child and the speed at which the child stopped running will be converted into VO_2_ max estimates.

##### Upper body strength

Upper body strength will be determined with the grip strength test, with both right and left hands. The Saehan hydraulic hand dynamometer (MSD Europe BVBA; Tisselt, Belgium) will be used for this test. Before the start of the test, the hand span (distance from the tip of the thumb to the tip of the little finger) of the child’s dominant hand will be measured (to the nearest 0.5 cm), and the grip span on the dynamometer will be adjusted accordingly [76, 77]. The outdoor staff will explain to the child how to hold the dynamometer correctly. The child, while sitting in an upright position, should grip the dynamometer with the arm fully extended. During this time, no other parts of the body should touch the dynamometer, and the arm being tested may not be squeezed against the body. Each child will then have three tries, or six tries in total (with a 30-s rest in between) to grip the dynamometer as hard as possible with alternating hands. The maximum reading, measured to the nearest 1 kg, will be recorded.

#### Parental survey

##### Dietary intake information

Information on dietary intake will be obtained from the parents/guardians to determine the adequacy of child’s intake of macro- and micronutrients. Dietary intake of children will be assessed using a culturally sensitive food frequency questionnaire (FFQ) for each country [78, 79]. The FFQ will be administered to the child’s carer at baseline.

##### Food insecurity

USAID (www.usaid.gov) defines food security as a situation in which all people at all times have physical and economic access to sufficient food to meet their dietary needs for a productive and healthy life. For the purpose of this study, food insecurity will be measured with a questionnaire based on the Household Food Insecurity Access Scale [80], which has been validated in industrialized countries and LMICs. For example, the study by Knueppel et al. [81] showed satisfactory validity and reliability among poor households in rural Tanzania.

##### Sleep

To gather information about children’s sleep duration, parents/guardians will report the time at which their child goes to bed and wakes up in the morning on school nights and school days.

##### Socioeconomic status

To estimate the SES, parents/guardians will be asked to answer nine items, covering household-level living standards, such as infrastructure and housing characteristics (house type, number of bedrooms, type of toilet and access to indoor water, indoor toilet/bathroom and electricity) and questions related to the ownership of three durable assets (presence of a working refrigerator, washing machine and car). The dichotomized items (0 = poor quality, not available; 1 = high quality, available) will be summed up to build an overall SES index, with higher scores reflecting higher SES. The validity of similar measures was established in previous research [82].

##### Distance from home to school

Distance from home to school and mode of traveling to/from school will be taken into consideration as a confounding variable. Therefore, we will ask the parents/guardians about their living place (township, village and area). Using Google maps, we will determine the distance between the residence and the school location.

### Statistical analyses

To assess the effects of the intervention, changes in outcome variables over the three time points will be analysed using repeated measures analyses of variances (ANCOVAs), with three between-subject factor groups (physical activity intervention: yes/no; multi-micronutrient intervention: yes/no; and weight status: underweight, normal weight, or overweight/obese) and a within-subject factor time (baseline, 9 months, and 21 months), after controlling for relevant confounders. To take into account the non-independence of the children within a class, we will examine between-cluster differences in potentially relevant confounders, which might include characteristics of the children (e.g. sex, socioeconomic status, and ethnicity), class (e.g. class size) and the intervention (e.g. compliance with intervention). If we find systematic and substantial differences between the clusters for these variables, the factors will be controlled as covariates. Moreover, we will screen the data for univariate and multivariate outliers before performing the main analyses in order to exclude students with unrealistically high or low values. In case of missing values (e.g. when participants drop out), all analyses will be performed with and without an intention-to-treat [38]. After a thorough dropout analysis, a decision will be reached regarding the best suited method to analyse intention-to-treat effects (e.g. imputation of missing values) [39]. If significant group or time interactions are present, Bonferroni-adjusted post-hoc tests will be performed to identify individual differences. Statistical significance level will be defined at an alpha level of 0.05. Effect size will be calculated according to the recommendations of Cohen [83], with 0.49 ≥ *d* ≥ 0.20 indicating small (negligible practical importance), 0.79 ≥ *d* ≥ 0.50 indicating medium (moderate practical importance) and *d* ≥ 0.80 indicating large effects (crucial practical importance). Additionally, bivariate and multivariate relationships between study variables will be tested using correlational analyses, regression analyses or structural equation modelling.

### Ethical considerations

#### Ethical approval and trial registration

The study will be carried out in accordance with the protocol and with principles in the current version of the Declaration of Helsinki and the guidelines of Good Clinical Practice (GCP) issued by the International Conference of Harmonisation (ICH).

The study can only begin once approval from the required authorities has been received. Any additional requirements imposed by the authorities shall be implemented. Ethical approval has been obtained from the ‘Ethikkommission Nordwest- und Zentralschweiz’ in Switzerland (EKNZ; reference number: Req-2018-00608). The intervention study has been registered in the ISRCTN registry (http://www.isrctn.com/ISRCTN29534081).

The responsible investigator at each site ensures that ethical approval from an appropriately constituted competent ethics committee (CEC) is sought for the clinical study. The study protocol has been approved in Côte d’Ivoire by the Institutional Review Board (IRB) of the Centre Suisse de Recherches Scientifiques en Côte d’Ivoire (CSRS; Abidjan, Côte d’Ivoire) and the Comité National d’Ethique et de la Recherche (CNER), reference number: 100–18/MSHP/CVESVS-km. Approval has also been obtained in South Africa from the research ethics committee of the Nelson Mandela University in Port Elizabeth (reference number: H18-HEA-HMS-006) and the Department of Education of the Eastern Cape Province. Moreover, the study protocol has been approved by the responsible ethics committee in Tanzania (Ifakara Health Institute (IHI–IRB), the National Institute for Medical Research (NIMR) and the Tanzania Food and Drugs Authority (TFDA).

The principal investigator (MG) and local project leaders (BB, CW, and HM) are allowed to amend the protocol or to provide suggestions for a protocol amendment. Should amendments to the approved protocol be required during the project, these would only be implemented after receiving the approval of the CEC.

#### Right to privacy and confidentiality

The principal investigator and local project leaders affirm and uphold the principle of the participants’ rights to privacy and that they will comply with applicable privacy laws. Anonymity of the participants will be guaranteed when presenting the data at national or international conferences or publishing key findings in the peer-reviewed literature. Individual participant medical information obtained as a result of this study will be considered confidential, and disclosure to third parties is prohibited. Participant confidentiality will be further ensured by utilizing participant identification code numbers to correspond to treatment data in the computer files. For data verification purposes, authorised representatives of the EKNZ and the respective Human Ethics Committee at site may require direct access to parts of the clinical records relevant to the study, including participants’ medical histories.

#### Referral to local clinics

Children who suffer from severe medical conditions and/or malnourishment (as diagnosed by a nurse, following national guidelines) will be referred to local clinics. If children are infected with malaria at the time of the data assessment, immediate treatment will be offered to the children.

#### Incentive for schools

As an incentive for schools, schools will be equipped with basic sports equipment and a music centre that allows for the implementation of moving-to-music lessons. Moreover, in South Africa, physical activity-friendly school environments will be developed through the implementation of simple painted games to encourage the free play of children.

#### Data processing and archiving

Data will be double-entered, checked, and merged into a single SPSS file. Survey data obtained from the paper and pencil questionnaire will be scanned and entered automatically (using EvaSys software). Data analysis will be performed with established software packages (e.g. SPSS, STATA and Mplus). SPSS syntax files will be documented to assure transparency of the conducted data analysis and to assure GCP.

Data will be saved electronically. Backup files will be stored regularly on the external cloud *Switchdrive@Universität Basel*. The personal data of the participants will be encrypted, and all the data obtained (e.g. hand-written paper questionnaires and the case report forms (CRFs)) will be used exclusively for scientific research. The local study leader will keep records in locked cupboards, and after 10 years, these records will be destroyed. Completed paper sheets will be locked and stored at the respective home institution. Electronic data files will be archived on the database *Switchdrive@Universität Basel*. Only authorised investigators will have access to data files. However, in line with the guidelines and open access policies of nationally and internationally recognised foundations and institutions, the published data from our project will be made publicly available (as Additional file 1).

#### Storage of biological material

Dried blood spots will be collected on filter paper and stored in a freezer at the home institution. Due to the complexity of assaying cytokines in dried blood spot samples, all samples will be shipped to the Global Clinical and Viral Laboratory in Durban, South Africa. Thus, biological samples from Côte d’Ivoire and Tanzania will leave the country conditional to material transfer agreements. Dried blood spot analyses will be carried out soon thereafter at the laboratory. Samples will be destroyed upon completion of all required assays.

#### Safety

Research assistants shall report all serious adverse reactions and adverse events (AEs) that might occur after deworming, finger pricking or multi-micronutrient supplementation immediately to the local study leader and principal investigator of the clinical trial. More specifically, during the entire duration of the study, all AEs and all serious adverse events (SAEs) will be collected, fully investigated and documented in source documents and CRFs. The study duration encompasses the time from when the first participant signs the informed consent until the last protocol-specific procedure has been completed, including a safety follow-up period. In case of any SAE, the participant’s well-being will be followed up until he or she shows normal laboratory values or vital signs below alert. Project leaders at the site are obliged to document and report the process, independently of the participant’s termination of study. Each research institution will ensure that the required insurance coverage is in place for the trial under applicable laws. In case of AEs or SAEs, unblinding of the participant is permissible.

#### Data monitoring and publication of data

The trial steering committee (composed of the sponsor-investigator (MG), the director of the Swiss Tropical and Public Health Institute (JU), and the principal investigators of the three study countries (BB, CW, HM)) will coordinate data monitoring, interim analysis, and dissemination of the study results through presentations at national and international conferences and publications in primarily open-access peer-reviewed journals. The trial steering committee will decide which researchers (beyond those listed as co-authors in the current study protocol) will have access to the final trial dataset. In agreement with the other members of the trial steering committee, the sponsor–investigator has the right to terminate the study prematurely according to certain circumstances, including ethical concerns, insufficient participant recruitment, and safety issues. Additional reasons for study termination include alterations in accepted clinical practice that render the continuation of the trial unwise and evidence of benefit or harm of the experimental intervention. On request of the local ethical review boards, an independent data monitoring committee will be appointed. The data monitoring board will have access to the unblinded interim data and can make recommendations to the trial steering committee. Trial auditing is carried out at the end of each year, whereas annual financial reports will be audited by an external auditor in each study country.

At the end of the study, the results will be communicated to the respective health and education authorities in the study countries and to the involved schools. All intervention materials will be made available to the respective control groups, so that the whole community can benefit from this project. Workshops will be offered to all teachers at the involved schools to assist with the implementation of the school-based health promotion programme.

### Schedule and milestones

Recruitment of participants started in Côte d’Ivoire on October 18, 2018. Follow-up assessments will be complete in March 2021. The project will end in December 2021. An approximate schedule is presented in Table 3.

**Table 3:** Planned schedule and milestones

Due date	Milestone title
April 30, 2018	Establishing contacts with authorities and schools
June 30, 2018	Development of intervention materials
December 31, 2018	Completion of initial teacher training in all countries
March 31, 2019	Completion of baseline (T1) data assessment in all countries
November 30, 2019	Completion of T2 follow-up data assessment in all countries
November 30, 2019	Completion of first year of intervention in all countries
November 30, 2020	Completion of T3 follow-up data assessment in all countries
March 31, 2021	Completion of second year of intervention in all countries
November 30, 2021	Completion of presentation of results to local authorities
December 31, 2021	Final report

## Discussion

This investigation has scientific relevance because it is among the first to explore the associations between objectively assessed physical activity/fitness, micronutrient deficiencies, body composition, infectious diseases, inflammatory and cardiovascular health risk markers and sleep indicators in schoolchildren in three African settings. Moreover, our study will contribute to a deeper understanding of the effects of a school-based intervention, combining physical activity and multi-micronutrient supplementation. The study will provide new insights in how schools can be empowered to implement school-based health promotion measures. Finally, as happens in many African countries, physical education and sport activities are not implemented as foreseen because academic subjects are considered more important, and this project could help to maintain or even increase the time allowed for physical activities in the schools. Time allocation is crucial along with empowerment of the actors.

Additionally, the study will provide important information about factors that may negatively affect the quality of the implementation of the intervention. Our study will also highlight the opportunities and challenges associated with capacity building in disadvantaged schools, to empower them to carry out health promotion measures with their own limited resources. Using accelerometer-based physical activity data will allow a more valid estimate of the level of physical activity of African schoolchildren inside and outside the school and will provide new insights into how a physical activity intervention impacts schoolchildren’s physical activity behaviour.

In addition, our research will provide a comprehensive update on the status of micronutrient deficiencies, inflammatory and cardiovascular health risk markers and communicable diseases in the selected communities in Port Elizabeth (South Africa), Ifakara (Tanzania), and Taabo (Côte d’Ivoire) that are at different stages of an epidemiological and nutrition transition. By linking these factors with cognitive and academic performance, children’s subjective health perceptions, and their psychosocial well-being, this wealth of information will reveal the true health consequences associated with the potential dual burden of diseases and will provide guidance for further health interventions to be implemented among school-aged children in these settings and elsewhere.

Thus far, only a few trials have investigated the potential of multi-micronutrient supplementations in African children. While physical activity was offered as part of our initial DASH study, the data assessment was limited to urban townships in South Africa. Hence, the current study will provide additional relevant information on whether or not the findings can be generalized to rural or peri-urban areas and to other African countries.

### Trial status

The study protocol corresponds to the first version of the protocol, as submitted to the EKNZ on July 19, 2018. The recruitment will start on October 18, 2018, in Côte d’Ivoire, in January 2019 in South Africa and in July 2019 in Tanzania. Data assessment should be complete in June 2020 in Côte d’Ivoire, in December 2020 in South Africa, and in March 2021 in Tanzania. Ethical approval has been obtained from the relevant review boards in Switzerland, Côte d’Ivoire, South Africa and Tanzania.

## Supplementary information

**Additional file 1:** Hypotheses associated with each of the study’s outcomes.

**Additional file 2:** Spirit flow chart.

## Data Availability

All data analysed during this study will be included in the published articles and their supplementary information files. The *KaziKidz* intervention material will be made publicly available on www.kazibantu.org.

## Abbreviations

AE: adverse event
ANCOVA: analysis of covariance
BIA: bioelectrical impedance analysis
BMI: body mass index
CDC: Centers for Disease Control and Prevention
CEC: competent ethics committee
CNER: Comité National d’Ethique et de la Recherche in Côte d’Ivoire
CSRS: Centre Suisse de Recherches Scientifiques en Côte d’Ivoire
DASH: Disease, Activity and Schoolchildren’s Health project
EKNZ: Ethikkommission Nordwest- und Zentralschweiz
FFQ: food frequency questionnaire
FGD: focus group discussion
GCP: good clinical practice
Hb: haemoglobin
HbA1c: glycated haemoglobin
HDL-C: high-density lipoprotein
HRQoL: health-related quality of life
ICH: International Conference of Harmonisation
IHI-IRB: Institutional Review Board of the Ifakara Health Institute
IL-6: human interleukin 6
ISRCTN: International Standard Randomized Controlled Trial Number
KIDSCREEN-10: 10-item instrument to assess health-related quality of life among children
LDL-C: low-density lipoprotein
LMICs: low- and middle-income countries
NCDs: non-communicable diseases
PAQ-C: physical activity questionnaire for children
POC: point-of-care
PSQI: Pittsburgh sleep quality index
RCT: randomized controlled trial
SAE: serious adverse event
SD: standard deviation
SES: socioeconomic status
SPSS: Statistical Package for the Social Sciences
STATA: statistics and data software
TC: total cholesterol
TFDA: Tanzania Food and Drugs Authority
TG: triglycerides
USAID: US Agency for International Development
WHO: World Health Organization
