# Supplementary material for: Effects of school-based physical activity and multi-micronutrient supplementation intervention on growth, health and well-being of schoolchildren in three African countries: the KaziAfya cluster randomised controlled trial protocol with a 2 × 2 factorial design
Source: Trials. 2020 Jan 6;21:22. doi: 10.1186/s13063-019-3883-5 (PMC6945709; doi:10.1186/s13063-019-3883-5)
Supplement: Supplementary file 1 — Additional file 1: Hypotheses associated with each of the study’s outcomes. [file 13063_2019_3883_MOESM1_ESM.docx]

***Additional file 1***

**Effects of school-based physical activity and multi-micronutrient supplementation intervention on growth, health and wellbeing of schoolchildren in three African countries: the *KaziAfya* cluster randomised controlled trial protocol with a 2x2 factorial design**

**Hypotheses**

In the following paragraphs, we provide an overview of the hypotheses associated with each of the study’s outcomes.

***a. Physical activity and fitness***

Based on previous research ([1](#_ENREF_1), [2](#_ENREF_2)), we expect that the total physical activity levels will increase among children who take part in a school-based physical activity intervention programme (particularly intra-curricular physical activity). We also assume that cardiorespiratory fitness will increase among children in the physical activity and physical activity plus multi-micronutrient supplementation condition, independent of children’s gender or weight status ([3](#_ENREF_3), [4](#_ENREF_4)).

***b. Micronutrient status***

There is extensive literature on the benefits of supplementation on improving micronutrient status and reducing deficiencies ([5](#_ENREF_5), [6](#_ENREF_6)). Accordingly, we expect that children in the multi-micronutrient supplementation group and children in the multi-micronutrient supplementation plus physical activity group will show significant improvements regarding their micronutrient status as well as a reduction in micronutrient deficiencies.

***c. Body Mass Index (BMI) and body composition***

While some school-based physical activity interventions have shown positive effects on children’s BMI and body composition ([7-9](#_ENREF_7)), the current state of research is inconclusive ([4](#_ENREF_4), [10](#_ENREF_10)). Effects seem to depend on children’s age, gender and type of intervention. However, in the DASH pilot study, we found a positive impact of the physical activity component on children’s BMI ([11](#_ENREF_11)). We therefore expect that the physical activity intervention might be associated with a decreased BMI in overweight or obese children. Additionally, a number of studies have examined the effect of micronutrient supplementation on body weight and body composition. Multivitamin and multi-mineral supplementation of obese Chinese women showed that supplementation could reduce body weight and fatness and improve serum lipid profiles, possibly through increased energy expenditure and fat oxidation ([12](#_ENREF_12)). Arsenault et al. ([13](#_ENREF_13)) reported that children with initial mild-to-moderate stunting who received a zinc supplement had a greater increase in fat-free body mass than those who did not receive additional zinc. In animal models, micronutrient supplementation has been associated with decreased adiposity ([14](#_ENREF_14)). Therefore, it is expected that children in the multi-micronutrient supplementation intervention and multi-micronutrient supplementation plus physical activity intervention arm will show a decrease of fat mass.

***d. Leptin***

Studies have reported that vitamin A, vitamin A precursors, and vitamin D down-regulate leptin through nuclear hormone receptors and have been found to up-regulate adiponectin expression. Zinc deficiency leads to reduced serum leptin concentration in rats and humans, and reduced leptin secretion by rat adipocytes, while repletion reversed this effect ([15-17](#_ENREF_15)). Based on these findings, we hypothesize that children in the multi-micronutrient supplementation and multi-micronutrient supplementation plus physical activity groups will have reduced serum leptin concentration and increased adiponectin concentrations.

***e. Inflammatory markers***

Research with adults showed that increased physical activity is associated with lower levels of inflammatory markers ([18](#_ENREF_18)) and that regular exercise training is associated with decreases in IL-6 ([19](#_ENREF_19)). Similar effects were found among children ([20](#_ENREF_20)). Particularly positive effects are to be expected among overweight/obese children ([21](#_ENREF_21)). Given these observations, we expect positive effects of physical activity and physical activity plus multi-micronutrient supplementation on IL-6. Additionally, supplementation trials have generally reported that single micronutrients have a beneficial effect on serum concentration of inflammatory markers. Vitamin A supplementation, for example, reduces serum concentrations of inflammatory markers in both obese and non-obese reproductive age women ([22](#_ENREF_22)). Levels of IL-6 were found to be significantly decreased with zinc supplementation, but not in the placebo group among obese women ([23](#_ENREF_23)). Vitamin D was reported to improve adipose tissue oxidative stress and inflammatory parameters in obese rats ([22](#_ENREF_22)). These findings suggest that children in the multi-micronutrient supplementation, physical activity, and multi-micronutrient supplementation plus physical activity arms will show reduced IL-6 levels after completion of the intervention programme.

***f. Cardiovascular health risk markers***

Among children, favourable effects of exercise interventions on resting blood pressure ([24](#_ENREF_24)) and blood lipids ([25](#_ENREF_25)) are well established, a relationship which is also observed among overweight and obese children ([26](#_ENREF_26)). Moreover, among children, several authors highlighted that features of the metabolic syndrome are associated with insufficient physical activity levels ([27-30](#_ENREF_27)). By contrast, the efficacy of multi-micronutrient supplementation on cardiometabolic risk markers reported in randomised clinical trials has not been consistent, and most of these trials have been carried out in high-income countries. A study carried out among overweight and obese Chinese women reported that multi-micronutrient supplementation was associated with improvements in lipid profiles ([12](#_ENREF_12)). Given this background, we hypothesize that physical activity, multi-micronutrient supplementation, and physical activity plus multi-micronutrient supplementation will result in decreased blood pressure, improved blood lipid profiles, and decreased blood glucose levels.

***g. Cognitive function and academic achievement***

Acute and regular physical activity and cardiorespiratory fitness are associated with better cognitive performance and academic achievement among children ([31-34](#_ENREF_31)). Additionally, a strong relationship has been found between moderate-to-severe anaemia or chronic anaemia and lower mental and psychomotor development scores. Scholastic achievement is also lower and anaemic children are twice more likely to have problems with mathematics ([35](#_ENREF_35)). Iron supplementation improves mental development scores modestly. This effect is particularly apparent for intelligence tests above 7 years of age and in initially anaemic or iron-deficient anaemic subjects ([36](#_ENREF_36)). Scholastic performance and aspects of executive function were also found to be improved among 7-year-old Bangladeshi children when antenatal or newborn supplementation with vitamin A were provided ([37](#_ENREF_37)). These findings lead us to expect that children in the physical activity, multi-micronutrient supplementation, and the physical activity plus multi-micronutrient supplementation groups will have increased cognitive function and academic performance compared to controls.

***h. Health-related quality of life and school-based stress***

Previous research has shown that school-based physical activity programmes positively impact on children’s well-being ([38](#_ENREF_38), [39](#_ENREF_39)). The developed physical activity program was designed towards the activity preferences of young children, and was highly enjoyed by children of the pilot testing. We therefore expect that children assigned to the physical activity and physical activity plus multi-micronutrient supplementation arms will improve in terms of health-related quality of life and perceived school-related stress. The expected benefits of multi-micronutrient supplementation across the different health and cognition outcomes ([40](#_ENREF_40), [41](#_ENREF_41)) also lead us to expect that children in the multi-micronutrient supplementation, and multi-micronutrient supplementation plus physical activity arms will have increased health-related quality of life and well-being.

***i. Sleep***

Research among pediatric samples showed that sleep alterations are highly prevalent among children with iron deficiency anemia ([42](#_ENREF_42)). Moreover, extensive literature has shown that increased levels of physical activity positively impact on sleep quality ([43](#_ENREF_43)). Based on these findings we expect better perceived sleep quality among children of the multi-micronutrient supplementation, physical activity, and multi-micronutrient supplementation plus physical activity groups, compared to controls. Sleep also plays an important role in the pre-level of inflammatory markers, such as CRP and IL-6 ([44](#_ENREF_44)).

***j. Reinfection rate with parasitic infections***

There is compelling evidence that parasitic diseases are intimately linked to conditions of poverty ([45](#_ENREF_45)). Hence, reinfection occurs rapidly after treatment intervention in the absence of complementary control and prevention measures ([46-48](#_ENREF_46)). We therefore hypothesize that well-nourished children are at lower risk of reinfection with parasites. It follows that children in the multi-micronutrient supplementation condition are thought to have slower reinfection rates with parasites. On the other hand, previous research has shown that due to increased exposure children with high physical activity levels might be at higher risk for parasitic infections ([49](#_ENREF_49)). It is therefore likely that the physical activity intervention is associated with a higher reinfection rate.

***k. Further hypotheses***

Further hypotheses concern the potential mechanisms underlying the intervention effects as well as quantitative versus qualitative malnutrition. With regard to the potential mechanism underlying the intervention effects, cytokine assays are used to determine if the effect of multi-micronutrient supplementation on leptin and inflammatory cytokines may be a mechanism underlying the effect of supplementation on overweight/obesity. These responses, which are elevated among overweight and obese individuals, may play a role in regulating adiposity. Leptin, which regulates food intake and energy expenditure also regulates the constancy of adipose tissue ([50](#_ENREF_50)). Leptin is also part of chronic inflammation response where there is increased expression and release of the pro-inflammatory cytokines interleukin-6 (IL-6) and tumor necrosis factor-a (TNF-α) ([51](#_ENREF_51)). Deficient levels of the anti-oxidants, vitamin A, and zinc may alter the genetic expression of leptin and increase the risk of greater adiposity ([16](#_ENREF_16), [17](#_ENREF_17), [52](#_ENREF_52)). Changes in micronutrient status may also regulate the inflammatory response through the regulation of the leptin response. However, the direction of causality in these associations is not clear in population studies. We will therefore test the hypothesis that these responses would be reduced in the multi-micronutrient supplementation and the multi-micronutrient supplementation plus physical activity arms indicating that micronutrient status regulates such responses and so may underlie any changes found in adiposity in the intervention arms. This effect may be found among mild-to-moderately malnourished children as well because zinc supplementation was associated with greater increases in fat-free mass among mild-to-moderately stunted children in Peru ([13](#_ENREF_13)). If these changes are found to be associated with changes in adiposity then that would provide further support for the causal role of micronutrient status in determining body composition and the risk of overweight. As such, identification of specific physiological pathways involved in these relationships would provide a more complete understanding of how these responses interact. This insight may enable the development of micronutrient interventions that are more effective in reducing adiposity and increasing lean body mass when combined with physical activity.

Children with low-calorie intake or low protein-energy malnutrition also have underlying micronutrient deficiencies ([53](#_ENREF_53)). In fact, the associations between low protein-energy intake and poor health outcomes such as stunting or impaired growth are a result of underling micronutrient deficiencies. It is now clearly established through individual randomised controlled trials ([54](#_ENREF_54)) and meta-analyses ([5](#_ENREF_5)) of these studies that zinc deficiency is one of the primary causes of growth impairment rather than protein-energy malnutrition. Supplementation with zinc reduces growth impairment, especially among children who are the most severely stunted. Iron deficiency is widely prevalent in many low- and middle-income countries, including the three countries where the study is to be carried out. A randomised controlled trial concerned with the efficacy of iron supplementation on cognition among school-aged children in a South African rural community where stunting and mild stunting was prevalent did find significant improvements in verbal and non-verbal learning ([55](#_ENREF_55)). Deficiencies of the other micronutrients that are to be included in the supplement are prevalent in communities where protein-energy malnutrition is prevalent ([56](#_ENREF_56), [57](#_ENREF_57)). As a result, we expect that the supplements will have an impact on cognition and physical activity as well on the other outcomes in the three study sites and that these effects may be greatest among children who are qualitatively malnourished due to the presence of micronutrient deficiencies.

**References**

1. Dishman RK, Motl RW, Saunders R, Felton GM, Ward DS, Dowda M, et al. Enjoyment mediates effects of a school-based physical activity intervention. Med Sci Sport Exerc. 2005;37:478-87.

2. Dobbins M, Husson H, DeCorby K, LaRocca RL. School-based physical activity programs for promoting physical activity and fitness in children and adolescents aged 6 to 18. Cochrane Database Syst Rev 2013;2:doi:10.1002/14651858.CD007651.pub2.

3. Meyer U, Roth R, Zahner L, Gerber M, Puder J, Hebestreit H, et al. Contribution of physical education to overall physical activity. Scand J Med Sci Sport. 2013;23:600-6.

4. Thivel D, Isacco L, Lazaar N, Aucouturier J, Ratel S, Doré E, et al. Effect of a 6-month school-based physical activity program on body composition and physical fitness in lean and obese schoolchildren. Eur J Ped. 2011;170:1435-43.

5. Brown KH, Peerson JM, Rivera J, Allen LH. Effect of supplemental zinc on the growth and serum zinc concentrations of prepubertal children: A meta-analysis of randomized controlled trials. Am J Clin Nutr. 2002;75:1062-71.

6. Gera T, Sachdev HP, Nestel P, Sachdev SS. Effect of iron supplementation on hemoglobin response in children: systematic review of randomised controlled trials. J Pediatr Gastroenterol Nutri; 44:468-486). J Ped Gastroenterol Nutr. 2007;44:468-86.

7. Brown T, Summerbell C. Systematic review of school-based interventions that focus on changing dietary intake and physical activity levels to prevent childhood obesity: an update to the obesity guidance produced by the National Institute for Health and Clinical Excellence. Obes Rev. 2009;10:110-41.

8. Li X-H, Lin S, Guo H, Huang Y, Wu L, Zhang Z, et al. Effectiveness of a school-based physical activity intervention on obesity in school children: a nonrandomized controlled trial. BMC Public Health. 2014;14:doi: 10.1186/471-2458-14-1282.

9. Meyer U, Schindler C, Zahner L, Ernst D, Hebestreit H, van Mechelen W, et al. Long-term effect of a school-based physical activity program (KISS) on fitness and adiposity in children: A cluster-randomized controlled trial. PLoS One. 2014;9:e87929.

10. Harris KC, Kuramoto LK, Schulzer M, Retallack JE. Effect of school-based physical activity interventions on body mass index in children: A meta-analysis. CMAJ. 2009;180:719-26.

11. Müller I, et al. Effect of a multidimensional fitness education and hygiene intervention programme on physical fitness and body-mass-index in disadvantaged primary schoolchildren (DASH) in Port Elizabeth, South Africa: a longitudinal study. in revision.

12. Li Y, Wang C, Zhu K, Feng RN, Sun CH. Effects of multivitamin and mineral supplementation on adiposity, energy expenditure and lipid profiles in obese Chinese women. Int J Obes. 2010;34:1070-7.

13. Arsenault JE, López de Romaña D, Penny ME, Van Loan MD, Brown KH. Additional zinc delivered in a liquid supplement, but not in a fortified porridge, increased fat-free mass accrual among young Peruvian children with mild-to-moderate stunting. J Nutr. 2008;138:108-14.

14. Tallman DL, Taylor CG. Effects of dietary fat and zinc on adiposity, serum leptin and adipose fatty acid composition in C57BL/6J mice. J Biochem. 2003;14:17-23.

15. Chen MD, Song YM, Lin PY. Zinc effects on hyperglycemia and hypoleptinemia in streptozotocininduced diabetic mice. Horm Metab Res. 2000;32:107-9.

16. Mantzoros CS, Prasad AS, Beck FWJ, Grabowski SM, Kaplan J, Adair C, et al. Zinc may regulate serum leptin concentrations in humans. J Am Coll Nutr. 1998;17:270-5.

17. Menedez C, Lage M, Peino R, Baldelli R, Concheiro P, Dieguez C, et al. Retenoic acid and vitamin D powerfully inhibit in vitro leptin secretion by human adipose tissue. J Endocrinol. 2001;170:425-31.

18. Hamer M, Steptoe A. Walking, vigorous physical activity, and markers of hemostasis and inflammation in healthy men and women. Scand J Med Sci Sport. 2008;18:736-41.

19. Gondim OS, Nunes de Camargo VT, Gutierrez FA, Fatima de Oliveira Martins P, Pereira Passos ME, Momesso CM, et al. Benefits of regular exercise on inflammatory and cardiovascular risk markers in normal weight, overweight and obese adults. PLoS One. 2015;10:doi: 10.1371/journal.pone.0140596.

20. Roberts CK, Izadpanah A, Angadi SS, Barnard RJ. Effects of an intensive short-term diet and exercise intervention: comparison between normal-weight and obese children. Am J Physiol. 2013;305:R552-R7.

21. Coimbra S, Catarino C, Nascimento H, Alves AI, Medeiros AF, Bronze-da-Rocha E, et al. Physical exercise intervention at school improved hepcidin, inflammation, and iron metabolism in overweight and obese children and adolescents. Ped Res. 2017:doi:10.1038/pr.2017.139.

22. Farhangi MA, Mesgari-Abbasi M, Hajiluian G, Nameni G, Shahabi P. Adipose Tissue Inflammation and Oxidative Stress: the Ameliorative Effects of Vitamin D. Inflammation. 2017:doi: 10.1007/s10753-017-0610.

23. Kim JH, Ahn J. Effect of zinc supplementation on inflammatory markers and adipokines in young obese women. Biol Trace Elem Res. 2014;157:101-6.

24. Kelley GA, Kelley KS, Tran ZV. The effects of exercise on resting blood pressure in children and adolescents: A meta-analysis of randomized controlled trials. Prev Cardiol. 2003;6:8-16.

25. Stoedefalke K. Effects of exercise training on blood lipids and lipoproteins in children and adolescents. J Sport Sci Med. 2007;6:313-8.

26. Luo B, Yang Y, Nieman DC, Zhang Y, Wang J, Wang R, et al. A 6-week diet and exercise intervention alters metabolic syndrome risk factors in obese Chinese children aged 11–13 years. J Sport Health Sci. 2013;2:236-41.

27. Andersen LB, Harro M, Sardinha LB, Froberg K, Ekelund U, Brage S, et al. Physical activity and clustered cardiovascular risk in children: A cross-sectional study (The European Heart Study). Lancet. 2006;368:299-304.

28. Brage S, Wedderkopp N, Ekelund U, Franks PW, Wareham NJ, Andersen LB, et al. Features of the metabolic syndrome are associated with objectively measured physical activity and fitness in children: The European Youth Heart Study (EYHS). Diabetes Care. 2004;27:2141-8.

29. Ekelund U, Anderssen S, Andersen LB, Riddoch CJ, Sardinha LB, Luan J, et al. Prevalence and correlates of the metabolic syndrome in a population-based sample of European youth. Am J Clin Nutr. 2008;89:90-6.

30. Ekelund U, Luan J, Sherar LB, Esliger D, Griew P, Cooper A, et al. Moderate to vigorous physical activity and sedentary time and cardiometabolic risk factors in children and adolescents. JAMA. 2012;307:704-12.

31. Davis CL, Tomporowski PD, Boyle CA, Waller JL, Miller PH, Naglieri JA, et al. Effects of aerobic exercise on overweight children’s cognitive functioning. Res Quart Exerc Sport. 2007;78:510-9.

32. Etnier JL, Chang Y-K. The effect of physical activity on executive function: a brief commentary on definitions, measurement issues, and the current state of the literature. J Sport Exerc Psychol. 2009;31:469-83.

33. Etnier JL, Nowell PM, Landers DM, Sibley BA. A meta-regression to examine the relationship between aerobic fitness and cognitive performance. Brain Res Rev. 2006;52:119-30.

34. Ludyga S, Gerber M, Brand S, Holsboer-Trachsler E, Pühse U. Acute effects of moderate aerobic exercise on specific aspects of executive function in different age and fitness groups: A meta-analysis. Psychophysiology. 2016;53:1611-26.

35. Madan N, Rusia U, Sikka M, Sharma S, Shankar N. Developmental and neurophysiologic deficits in iron deficiency in children. Ind J Ped. 2011;78:58-64.

36. Sachdev HP, Gera T, Nestel P. Effect of iron supplementation on mental and motor development in children: systematic review of randomised controlled trials. Public Health Nutr. 2005;8:117-32.

37. Ali H, Hamadani J, Mehra S, Tofail F, Hasan MI, Shaikh S, et al. Effect of maternal antenatal and newborn supplementation with vitamin A on cognitive development of school-aged children in rural Bangladesh: A follow-up of a placebo-controlled, randomized trial. Am J Nutr. 2017;106:77-87.

38. Hartmann T, Zahner L, Puhse U, Schneider S, Puder JJ, Kriemler S. Physical activity, bodyweight, health and fear of negative evaluation in primary school children. Scand J Med Sci Sports. 2010;20(1):e27-34.

39. Rafferty R, Breslin G, Brennan D, Hassan D. A systematic review of school-based physical activity interventions on children’s wellbeing. Int Rev Sport Exerc Psychol. 2016;9:215-30.

40. Friis H, Mwaniki D, Omondi B, Muniu E, Thiong’o F, Ouma J, et al. Effects on haemoglobin of multi-micronutrient supplementation and multi-helminth chemotherapy: a randomized, controlled trial in Kenyan school children. Europ J Clin Nutr. 2003;57:573-9.

41. Kruger M, Gericke G, White Z. Micronutrients and bone growth in preadolescent children form developing countries. Open Nutr J. 2018;12:13-22.

42. Peirano PD, Algarin CR, Chamorro RA, Reyes SC, Duran SA, Garrido MI, et al. Sleep alterations and iron deficiency anemia in infancy. Sleep Med. 2010;11:637-42.

43. Lang C, Kalak N, Brand S, Holsboer-Trachsler E, Pühse U, Gerber M. The relationship between physical activity and sleep from mid adolescence to early adulthood. A systematic review of methodological approaches and meta-analysis. Sleep Med Rev. 2016;58:32-45.

44. Aragon-Arreola JF, Moreno-Villegas CA, Armienta-Rojas DA, D’e la Herran-Arita AK. An insight of sleep disorders in Africa. eNeurologicalScience. 2016;26:37-40.

45. King CH. Parasites and poverty: The case of schistosomiasis. Acta Trop. 2010;113:95-104.

46. Grimes JET, Croll D, Harrison WE, Utzinger J, Freeman MC, Templeton MR. The relationship between water, sanitation and schistosomiasis: A systematic review and meta-analysis. PLoS Negl Trop Dis. 2014;8(e3296): doi:10.1371/journal.pntd.0003296.

47. Jia T-W, Melville S, Utzinger J, King CH, Zhou X-N. Soil-transmitted helminth reinfection after drug treatment: A systematic review and meta-analysis. PLoS Negl Trop Dis. 2012;6:doi: 10.1371/journal.pntd.0001621.

48. Strunz EC, Addiss DG, Stocks ME, Ogden S, Utzinger J, Freeman MC. Water, sanitation, hygiene, and soil-transmitted helminth infection: A systematic review and meta-analysis. PLoS Med. 2014;11(e1001620):doi:10.1371/journal.pmed.1001620.

49. Gerber M, Müller I, Walter C, Du Randt R, Adams L, Gall S, et al. Physical activity and dual disease burden among South African primary schoolchildren from disadvantaged neighbourhoods. Prev Med. 2018;112:104-10.

50. Halaas JL, Boozer C, Blair-West J, Fidahusein N, Denton DA, Friedman JM. Physiological response to long-term peripheral and central leptin infusion in lean and obese mice. PNAS. 1997;94:8878-83.

51. Greenberg AS, Obin MS. Obesity and the role of adipose tissue in inflammation and metabolism. Am J Clin Nutr. 2005;83:461-5.

52. Friedman JM. The function of leptin in nutrition, weight, and physiology. Nutr Rev. 2002;60:1-14.

53. Black RE, Allen LH, Bhutta ZA, Caulfield LE, de Onis M, Ezzati M, et al. Maternal and child undernutrition: global and regional exposures and health consequences. Lancet. 2008;371:243-60.

54. Kikafunda JK, Walker AF, Allan EF, Tumwine JK. Effect of zinc supplementation on growth and body composition of Ugandan preschool children: A randomized, controlled, intervention trial. Am J Clin Nutr. 1998;68:1261-6.

55. Baumgartner J, Smuts CM, Malan L, Kvalsvig J, van Stuijvenberg ME, Hurrell RF, et al. Effects of iron and n-3 fatty acid supplementation, alone and in combination, on cognition in school children: A randomized, double-blind, placebo-controlled intervention in South Africa. Am J Clin Nutr. 2012;96:1327-38.

56. Donnen P, Brasseur D, Dramaix M, Vertongen F, Ngoy B, Zihindula M, et al. Vitamin A deficiency and protein-energy malnutrition in a sample of pre-school age children in the Kivu Province in Zaire. Europ J Clin Nutr. 1996;50:456-61.

57. Walter EA, Scariano JK, Easington CR, Polaco AM, Hollis BW, Dasgupta A, et al. Rickets and protein malnutrition in northern Nigeria. J Trop Ped. 1997;43:98-102.
