# Supplementary material for: Effects of school-based physical activity and multi-micronutrient supplementation intervention on growth, health and well-being of schoolchildren in three African countries: the KaziAfya cluster randomised controlled trial protocol with a 2 × 2 factorial design
Source: Trials. 2020 Jan 6;21:22. doi: 10.1186/s13063-019-3883-5 (PMC6945709; doi:10.1186/s13063-019-3883-5)
Supplement: Supplementary file 2 — Additional file 2: Spirit flow chart. [file 13063_2019_3883_MOESM2_ESM.docx]

**Effects of school-based physical activity and multi-micronutrient supplementation intervention on growth, health and wellbeing of schoolchildren in three African countries: the KaziAfya randomised controlled trial protocol**

**Spirit Figure**

|  | Study period | | | | |
| --- | --- | --- | --- | --- | --- |
|  | Enrolment | Allocation | Post allocation | | |
| Timepoint | -t1 | 0 | T1 | T2 | T3 |
| Enrolment |  |  |  |  |  |
| Eligibility screen | X |  |  |  |  |
| Informed consent | X |  |  |  |  |
| Allocation |  | X |  |  |  |
| Interventions |  |  |  |  |  |
| Control |  |  | 0 mo | 9 mo | 21 mo |
| PA |  |  | 0 mo | 9 mo | 21 mo |
| MMNS |  |  | 0 mo | 9 mo | 21 mo |
| PA + MMNS |  |  | 0 mo | 9 mo | 21 mo |
| Asssessment |  |  |  |  |  |
| Clinical examination |  |  | X | X | X |
| Blood testing |  |  | X | X | X |
| Anthropometric measurements |  |  | X | X | X |
| Parasitological examinations |  |  | X | X | X |
| Cognitive function/academic performance |  |  | X | X | X |
| Student survey |  |  | X | X | X |
| Accelerometry |  |  | X | X | X |
| Fitness testing |  |  | X | X | X |
| Parental survey |  |  | X | X | X |

Notes. PA = Physical activity, MMNS = Multi-micronutrient supplementation
